# Experienced Effects on Well-Being following Smoking Cessation: Findings from the 2020 ITC Four Country Smoking and Vaping Survey

**DOI:** 10.3390/ijerph191610037

**Published:** 2022-08-15

**Authors:** Lin Li, Ron Borland, Hua-Hie Yong, Shannon Gravely, Geoffrey T. Fong, Kenneth Michael Cummings, Katherine East, Michael Le Grande

**Affiliations:** 1 School of Psychological Sciences, The University of Melbourne, Melbourne, VIC 3010, Australia; 2 School of Psychology, Deakin University, Geelong, VIC 3220, Australia; 3 Department of Psychology, University of Waterloo, Waterloo, ON N2L 3G1, Canada; 4 School of Public Health Sciences, University of Waterloo, Waterloo, ON N2L 3G1, Canada; 5 Ontario Institute for Cancer Research, Toronto, ON M5G 0A3, Canada; 6 Department of Psychiatry & Behavioral Sciences, Medical University of South Carolina, 67 President St., Charleston, SC 29425, USA; 7 Hollings Cancer Center, Medical University of South Carolina, 67 President St., Charleston, SC 29425, USA; 8 National Addiction Centre, Institute of Psychiatry, Psychology and Neuroscience, King’s College London, 4 Windsor Walk, Denmark Hill, London SE5 8BB, UK

**Keywords:** smoking cessation, ex-smokers, vaping, perceived effects, quality of life, coping with negative emotions, health concerns

## Abstract

Background and Aims: There has been limited research addressing changes in subjective well-being as a result of quitting smoking. This paper examines recent ex-smokers’ well-being related experiences overall and as a function of (1) duration of cessation and (2) continued nicotine use from vaping. Methods: A sample of 1379 ever-daily smoking ex-smokers (quit for up to 5 years) from the 2020 ITC Four Country Smoking and Vaping Survey (Australia, Canada, the UK, and the US), of which 27.1% currently vaped daily. Well-being measures were perceived changes post-quitting in emotion coping (stress and negative emotions), enjoyment of life, and day-to-day functioning. We also assessed the level of persisting worry about past smoking leading to future health problems. Results: Overall, among those answering all four well-being measures, 51.8% of the ex-smokers reported positive effects and no negatives, but 27.3% reported at least one negative effect, with the remainder reporting no change in any measure. Positive effects were greater among those who had quit more than 1 year prior. The largest improvement (56.3%) was for daily functioning, which showed improvement over time since having quit. Current daily vapers reported similar well-being as those not vaping; however, fewer daily vapers reported worsening ability to cope with stress (10.2% vs. 20.7%). Overall, 84% reported being worried about future negative health effects of smoking, with no clear differences by quitting duration or vaping status. Conclusions: Most ex-smokers reported changes in their well-being since quitting, with more reporting improvements than declines. Well-being improved with duration of time since quitting, but did not appear to be influenced by daily vaping use, but stress coping may be better among vapers. Persisting worries about possible future health effects from smoking may be reducing the experienced benefits of quitting smoking for some.

## 1. Introduction

The benefits of smoking cessation on health outcomes are well established (e.g., reduced risk of cardiovascular diseases and cancer) [1]. There is increasing evidence that cessation of smoking is associated with improvements in mental health, stress levels, and well-being [2,3]. This paper explores what aspects of well-being are improved and whether continued vaping modifies these effects.

People are unlikely to sustain attempts to stop smoking if they experience sustained decrements to their well-being, even if they accept the long-term risks of continuing to smoke [4]. People who smoke report experiencing pleasure and reduced stress and anxiety when they smoke [5,6,7]. They also report concerns about the effects of quitting smoking, such as weight gain, decreased ability to cope with stressors and negative affect, loss of pleasure, and difficulty coping with cravings [3,8].

Smoking abstinence can trigger symptoms of withdrawal from nicotine (e.g., irritability, depressed mood, restlessness, anxiety) and consequent cravings to smoke [9]. The negative immediate effects of smoking cessation on well-being can reinforce a perception that smoking is necessary to maintain a sense of normalcy and well-being. Even when the immediate experiences of quitting smoking are negative, how this is interpreted is also likely to affect relapse proneness. For example, consider two people quitting smoking who experience the same mild dysphoria. If one anticipates that mild dysphoria is normal for a short period following smoking abstinence, that person is more likely to persist in quitting than someone for whom the experience of mild dysphoria is unexpected and thus is used as an excuse to return to smoking in order to feel normal again.

Although smoking is a response to nicotine addiction, it is also a highly conditioned behavior where specific moods, situations, or settings are associated with the rewarding effects of nicotine, and the occurrence of these smoking-related moods/events can serve as cues to trigger relapse among those trying to quit [5]. Thus, it can be expected that after abstention from smoking, experiences of reduced well-being (e.g., due to withdrawal) [3] are likely to rebound within weeks of successfully quitting [8]. There is increasing evidence that quitting smoking results in improvement in a wide range of mental health and quality-of-life measures [2]. However, findings of heterogeneity of responses to cessation suggest that mental health outcomes related to cessation may be influenced by context as well as individual biology.

Research to date has found that negative experiences post-quitting can be a reliable predictor of relapse, particularly effects on emotion and coping with stressors. On the other hand, positive responses to cessation tend not to be associated with increased likelihood of smoking abstinence [9,10]. There is also evidence that the determinants of relapse change as a function of duration of cessation [11,12].

In our earlier work on quitting, we found that reports of various levels of well-being were high from very early in quitting attempts and the average level increased with time since quitting (subsequently referred to as “time quit”), often according to a logarithmic function of time, with most of the increase in the early days and asymptotic with time [12]. In one study, we found high reported overall quality of life, with 76% of those who had quit a year or more prior reporting improvements in their overall quality of life and only 2% reporting reductions [8]. In another study, we found a similar rate of increase for reported enjoyment of life, but a somewhat more rapid asymptote with emotion-coping measures, dealing with stress and negative emotions [10]. In that same study, we found that negative effects were predictive of relapse, but positive changes were not [10]. As might be expected, concerns over future health risks from past smoking gradually declined over time [9]. Controlling for time quit, reported improvements since quitting were not associated with reduced relapse, and there was weak evidence that reductions in experienced negative affect coping were associated with increased subsequent relapse.

Piper et al. compared successful quitters in a trial from those who failed and also found a similar pattern of results of high levels of reported positive effects and greater improvements in a range of well-being measures for quitters, most strongly those related to mental health [3]. Taken together, the evidence seems to strongly suggest that for most ex-smokers, at least after the initial months, quitting is associated with experienced improvements in well-being [3,9,10,12]. However, a minority continue to report decrements [3,8]. Some of these might be attributable to other events in the person’s life, so we have no clear sense as to how much of this, if any, is due to having quit smoking.

If smokers are overestimating negative effects or underestimating positive effects of quitting, it may actually increase their risk of relapse. Perceived changes in subjective well-being as well as actual experiences may compete with the undoubted benefits to longer-term health in influencing smokers’ interest in quitting [4]. More compelling evidence on the likelihood of early perceived benefits could be used to quell many smokers’ fears and might actually be used to encourage more quitting attempts.

Apart from the changes in the early days post-quitting, little is known as to what factors are associated with experiencing different effects of quitting. As some of the positively experienced effects of smoking are due to nicotine use, ongoing nicotine use might have an effect. However, this might be greater for forms of nicotine delivery designed to maximize positive experiences, rather than products like conventional nicotine-replacement products, which have been largely designed to minimize positive experiences of use. Some smokers vape nicotine to help them stop smoking, and many continue to vape after stopping smoking [13,14]. However, it is unclear how regular vaping post-cessation of cigarettes might affect post-cessation subjective experiences of health and well-being. Vaping provides an alternative source of nicotine, albeit perhaps less effectively than from a cigarette for some at least, but delivered in a similar way and therefore sharing some of the subjective effects of smoking tobacco, and thus may reduce negative experienced effects of quitting smoking. Nicotine is a stimulant, and for some people appears to have cognitive enhancement benefits [15], so maintenance of nicotine use post-quitting for as long as needed may be important in terms of helping the quitter feel normal and avoiding cognitive deficits that can occur upon withdrawal from nicotine.

Effects as a function of previous strength of people’s smoking habit might also affect their experiences post-quitting. More strongly dependent smokers may be likely to have higher immediate negative experiences (including strong urges to smoke and other withdrawal-related effects), so might be expected to report worse outcomes at least early on, but to the extent that smoking is adversely affecting functioning, they might be prone to report greater improvement over time.

Finally, most smokers who quit do so to protect their future health [16,17]. However, effects on future health cannot be directly experienced at the time of the quit attempt. We have theorized [4] that continued worry about whether health has indeed been improved by quitting smoking could be a trigger for relapse, as it reduces the experienced benefit of having quit when it occurs (i.e., failure to gain expected reduction in concern about future health). However, in our earlier study, we found no significant association with quitting, but still high levels of persisting concern about effects of past smoking [10].

We might expect those who find quitting hardest—and any who gain functional benefits from smoking or more likely the nicotine—would also be more likely to report either adverse effects or less positive effects of quitting. We are also interested to see if any effects are mitigated in part or fully by the use of alternative forms of nicotine, in this case vaping.

The aims of this paper are to attempt to replicate findings from previous research showing that reported experiences of positive effects following quitting smoking are more common over time since quitting than reported negative experiences following quitting and that reported positives peak several months after quitting [9,10,11,12]. We are primarily interested in changes in people’s perceptions, rather than their inferences about the cause of any changes, so ask about changes since quitting, rather than changes as a result of quitting. We also explore how nicotine vaping after smoking cessation may modify the experiences that people report once they stop smoking. If nicotine aids stress and affect management, we hypothesize that nicotine vaping after cessation will be associated with better ability to cope with stress and emotions. On the other hand, if vaping reduces the short-term positive health changes associated with quitting, we might expect vapers to be associated with a lower likelihood of reporting improved day-to-day functioning. We expect persistence of worry to be high after cessation and to decline over time quit, but have no expectation regarding how vaping might change this outcome.

## 2. Materials and Methods

### 2.1. Data Source and Participants

The analytic sample came from the Wave 3 (February–June 2022) of the International Tobacco Control Four Country Smoking and Vaping (ITC 4CV) Survey and included 1379 ever-daily smoking ex-smokers who had quit smoking cigarettes within the past 5 years (Australia: *n* = 199; Canada: *n* = 450; England: *n* = 359; US: *n* = 371). The ITC 4CV Survey is an online cohort study conducted in the above four countries. Respondents (adults aged ≥ 18 years) were recruited by commercial panel firms in each country as established cigarette smokers, recent ex-smokers (quit ≤ 2 years), or vapers (vape ≥ weekly), with ex-smokers of a longer duration retained in subsequent waves. We restricted the sample to ex-daily smokers, as we would not expect much impact on the dependent measures among those who were not smoking much. More details about the ITC 4CV surveys are reported elsewhere [18,19].

### 2.2. Measures

#### 2.2.1. Key Measures

Measures of experienced well-being were two measures of emotion coping and two more general measures:

*Stress coping:* “Since you quit, has your ability to calm down when you feel stressed or upset [changed]?”; and *Negative affect coping*: “Since you quit, has your ability to control feelings like anger, grumpiness, or annoyance [changed]?” In subsidiary analyses, we computed a combined index, given the two are closely related.

*Enjoyment of life*: “Since you quit, has your capacity to enjoy the simple pleasures of life [changed]?”

All three of these had response options: 3. “Improved,” 2. “Stayed the same,” 1. “Gotten worse,” and “Don’t know,” with Don’t know combined with “Stayed the same” and scored as indicated [10].

In addition, among those who had quit within the last 2 years, we asked about *Daily functioning*: “What effect, if any, has quitting cigarette smoking had on how you function across the day?” “Improved my daily functioning a lot” and “Improved a little” (combined and coded 3), “Had no effect” or “don’t know” (coded 2), and “Made my daily functioning a little worse” or “A lot worse” (coded 1).

We also asked about *Persisting worry*: “How worried are you that your past cigarette smoking may lead to major illnesses in the future?” with response options: 1. “Not at all worried/don’t know,” 2. “A little worried,” 3. “Moderately worried,” or 4. “Very worried.” This was a modified version of a question asked previously [20].

#### 2.2.2. Focal Covariates

*Time since quitting smoking* was coded as “≤3 months,” “4 months–1 year,” “>1–2 years” and “>2–5 years.”

*Vaping status* was coded as “current daily,” “current weekly,” “past regular (at least weekly in past),” and “past never regular (including never used).” Current daily vapers (hereafter referred to as “daily vaping quitters” or “vaping quitters”) were compared with all others (referred to as “other quitters”).

#### 2.2.3. Other Measures

*Sociodemographics* included country, sex (male, female), and age (18–24, 25–39, 40–54, 55 and older). Due to differences in income and educational systems across countries, only relative levels of education and income were used (low, moderate, high). Details about country-specific income and education levels are provided elsewhere [20].

*Previous cigarette consumption*: Number of cigarettes smoked per day at peak in the past (a proxy for previous nicotine dependence) was asked of ever-daily smokers via “Thinking back over the last 5 years to the time when you were smoking the most, on average, how many cigarettes did you usually smoke each day?” (coded as “1–9 cigarettes,” “10–19,” and “20+”).

*Strength of urges* to smoke was asked via “In general, how strong have urges to smoke been in the last 24 h?” (coded as “not felt the urge to smoke,” “slightly,” and “substantially,” which included “moderately,” “strong,” and “extremely strong”).

*NRT use:* Use of nicotine-replacement therapy (NRT) was coded as “daily user” vs. “all others” (which included not at all use/don’t know, up to 4–6 days a week).

*Perceived past (and current) health effects of smoking* was asked via “To what extent, if at all, has smoking cigarettes damaged your health?) (“not at all,” “just a little,” “a fair amount,” “a great deal,” and “don’t know”).

### 2.3. Data Analysis

All analyses were conducted using Stata version 15.0. [21] Frequencies (on unweighted data) and cross-tabulations were performed to examine the distributions of categorical variables by duration of time quit and vaping status. Chi-squared tests were used to test for any group differences. We also looked at interrelationships within measures of perceived effects of quitting through correlations of descriptive statistics. To examine the relationships between ordinal variables (e.g., worry about harms of smoking), Cramér’s V (a measure of effect size) and Kendall’s tau-b values were computed. Adjusted ordered logistic regression analyses were used to examine the associations between each of the five post-quitting experience variables with duration of time quit, vaping status, and all covariates listed above included. We also tested for interactions between duration of time quit and vaping status on each of the post-quitting experience variables, but did not find any significant interaction effect. We also conducted a range of sensitivity analyses(see more details in the Section 3). In all analyses, a *p* value <0.05 was considered statistically significant.

## 3. Results

Table 1 presents the characteristics of the overall sample (*n* = 1379, of which 374 or 27.1% currently vaped daily), stratified by duration of time quit. There were few differences, but those aged 55 and older and daily vaping quitters were more likely to have quit smoking 2–5 years ago and those using NRT daily most likely to have quit less than 3 months ago.

As Table 2 indicates, the four measures of experienced well-being were all at least moderately positively correlated (between 0.35 and 0.69), with the two emotion-coping measures (stress coping and negative affect coping) most strongly correlated. By contrast, the question about persisting worry about damage to health from past smoking was only weakly but unexpectedly significantly positively correlated with three of the four well-being measures.

Table 3 presents perceived effects of quitting by duration of time quit and vaping status. In preliminary analyses, we also explored possible interactive effects of time quit for both vaping and NRT use, and as we found no significant effects, report the analyses without the interaction term included. Around a quarter reported improvements in negative affect coping, and almost half reported improvements in stress coping, which was much higher than reported declines. Almost 60% reported no effects for either measure. Nearly half (47.9%) reported improved capacity to enjoy life, and a majority (56.3%) reported that their day-to-day functioning was improved, with few reporting declines for either measure. For all four well-being measures, there were increases in positive reported effects relative to negative effects from 0–3 months to longer time quit, but there was no evidence of increases beyond 1 year. The coping measures changed from more negative to more positive and the other measures became even more net positive. There was no significant relationship with time quit for persisting worry.

Table 4 shows factors associated with ex-smokers’ perceived effects of quitting when controlling for demographics and the other variables listed in the table. Positive associations were found between duration of time quit and for the four measures of perceived effects of quitting, all improved beyond 3 months’ time quit (although stress coping was no longer significant), with the differences most evident after 1 year. There was no time-quit relationship with persisting worry. “Daily vaping quitters” continued to be more likely to report improved capacity to cope with stress (adjusted OR (aOR) = 1.30, 95% CI 1.01–1.66), but the positive relationship with coping with negative feelings was no longer significant (aOR = 1.28, 95% CI 0.99–1.65). Like the bivariate results, vaping status was not significantly associated with capacity to enjoy life or day-to-day functioning. The lack of relationship between vaping status and continued worry persisted.

Additional analyses (which were not included in any table) show that there were no significant differences by NRT use status (daily use vs. all other) for any of the four well-being measures. Overall, among those who had quit less than 2 years ago (i.e., those who were asked all four well-being measures), 51.8% reported at least one positive effect and no negative effects, 27.3% reported at least one negative effect, and the remaining 20.8% reported no changes on any of the four well-being outcomes. Positive effects increased with time since quitting (*p* < 0.05), and among those who had quit 1–2 years ago (*n* = 302), 57.3% reported positive effects and no negative effects, 23.2% any negatives, and 19.5% no changes on any of the four measures. Among those who had quit more than 2 years ago (*n* = 532), of the three questions asked, 49.0% reported positive effects and no negative effects, 20.9% any negatives, and 30.1% no changes.

A few demographic variables were related to the well-being measures (see Table 4). Those from Canada were most likely to report net positive effects on all four well-being measures. Females were less likely to report improved stress coping and negative affect coping, and were more likely to report residual worry. Compared with those aged 55 and above those aged 40–54 in particular were more likely to report improved day-to-day functioning and improved enjoyment of life, but more persistent worry. Age was unrelated to the two emotion-coping measures.

Past smoking of more than 10 cigarettes per day was associated with lower levels of improvement in the two emotional-coping measures, and current urges to smoke associated with reduced perceived benefit in all four well-being measures, but not for persistent worry. Current daily use of NRT was not related to any of the measures.

Reporting that past smoking had adversely affected health was positively associated with improvements in all four well-being measures and unsurprisingly very strongly positively associated with persistent worry about adverse effects of past smoking.

We conducted four sensitivity analyses. In one, we removed adverse effects of past smoking from the regressions and it did not affect the overall results, except that the higher level of residual worry in Canada became clearly non-significant. We also restricted the sample to ex-smokers who had quit more than 3 months ago (*n* = 1109), and the results were essentially the same. To better understand any potential impacts of persisting worry, we found that, the more worried the ex-smokers were, the more tired they were of staying quit (Cramér’s V = 0.09, Kendall’s tau-b = 0.10, ASE = 0.03, *p* = 0.001), but persisting worry was not associated with confidence in ability to stay quit (Cramér’s V = 0.07, Kendall’s tau-b = −0.008, ASE = 0.02, *p* = 0.13). Finally, we confirmed that there were no significant changes in Table 4 results when we excluded “Don’t know” well-being responses compared to recoding into “Stayed the same,” as presented in the table.

We also analyzed the composite coping measure and found both a significant bivariate and multivariate effect of greater positive effects or less negative among the vapers, both on univariate analysis (OR = 1.24, 95% CI 1.00–1.54, *p* = 0.048) and marginally more significant on multivariate analysis (aOR = 1.34, 95% CI 1.06–1.69, *p* = 0.015), with the daily vapers less likely to report negative effects.

## 4. Discussion

Consistent with previous studies [3,8,9,11] most ex-smokers in our study reported changes in their well-being since quitting, with more reporting improvements rather than declines. We extended this work to similar findings for perceived day-to-day functioning, and indeed this was the measure showing the highest level of positive change. Over half of the ex-smokers in our study reported at least one positive effect on well-being with no negative effects, and only around a quarter reported at least one negative effect. Overall, reported well-being increased with duration of time since quitting up to a year at least. The impact of continuing to vape on experiences was found only for stress coping, which appeared to be better among those continuing to vape after smoking cessation.

In contrast to the findings on well-being, most ex-smokers reported some level of persistent worry about effects of past smoking, and this continued unchanged over the period of abstinence studied. A greater proportion of ex-smokers reported improvements than declines in enjoyment of life, daily functioning, and ability to cope with both stress and negative emotions. Effects generally improved from the first 3 months, with no clear evidence of any improvements beyond 1 year quit, consistent with most of the changes in experienced effects occurring in the first weeks and months after quitting [3,8,10,12]. Improvements in coping (particularly negative affect copying) were less commonly reported and the differential between positives and negatives much smaller than that for both enjoyment of life and daily functioning. Those reporting reductions in emotion-focused coping declined with time quit such that those beyond 3 months quit reported more improvements than reductions. As these affective reactions are the areas where popular belief has it that smoking helps, these findings, along with others, that find essentially the same result [3,8,10,11,12,22] indicate that for most smokers, quitting is positive for emotional coping and perhaps coping overall, although there is a minority for whom it continues to cause problems in the longer term. Indeed, about 60% thought quitting had no impact on either. Our analyses suggest those who previously smoked more and those with persisting cravings are less likely to perceive benefits and consequently more likely to experience negative effects. We accept we do not know if the better reported outcomes beyond 3 months are due to changes in experiences within individuals or to those with negative early experiences being more likely to relapse. Given that negative experiences are a known determinant for relapse [23], at least part of the effect is likely to be due to differential relapse, although we have not tested it here.

In interpreting the findings, it is important to keep in mind that our measures are of perceived changes and might not reflect actual changes in the functions asked about. However, perceived changes are the evidence that people use in interpreting the likely benefits or costs of change, so they have potential to influence outcomes, at least partly independent of the underlying functioning. That said, over time, if the two are discordant, we would expect the perceptions to shift towards the underlying functions, as this is what determines actual functioning. For example, if you think your ability to cope with stress has improved, but when stressed your experience of coping does not seem to improve, you are likely to revise your perception that quitting smoking has had no effect. That the effects we report increased with time and there was no evidence of decline for any is consistent with the beliefs being concordant with the underlying functions.

The finding of a preponderance of benefits over losses is important, because it points to net perceived benefits of quitting smoking for most, which can be used as evidence to encourage more smokers to quit, along with the substantial evidence on reductions in actual diseases [1]. While the latter is intellectually compelling, it lacks direct experiential force [4], unless related improvements are experienced and related to the longer-term effects [1]. Consideration needs to be given to the minority who report decrements, which are particularly around emotion-focused coping. Some of these decrements may be due to extraneous factors (i.e., other life changes or stressors), but the fact that some appear to be attributed to quitting suggests some are making (or perceive themselves as making) some sacrifices to maintain abstinence.

While the benefits that occur are a positive outcome, other research suggests that unless the ex-smoker actually thinks about these benefits when at risk of relapse, these positive effects do not protect against relapse [4,11], so encouraging ex-smokers who experience gains to focus on what they are gaining when confronting urges to smoke may be a useful relapse-prevention strategy. A similar strategy might be applied to those who experience no negatives, as this is also important and should help protect against relapse, albeit less potently.

The findings on vaping are novel. Daily vaping was associated with perceptions of better capacity to deal with stress. It is plausible that vaping helps former smokers to manage their stress. Many smokers believe that smoking does, and the nicotine is the most plausible biological mechanism, as it is known to positively affect some cognitive and attentional processes [15]. However, it is important to canvass alternative explanations. First, vapers may be keen to promote their choice of vaping in the face of opposition, so may be prone to overstate benefits. This is plausible, but if it were so, we might expect a generalized positive evaluation. However, we found differential responding, with no greater claimed benefits outside coping. Further, we think it less likely to have vapers interpreting the questions as an evaluation of vaping by asking for change, rather than the additional cognitive step of attribution of causality for any experienced change. Regardless of the underlying reality, daily vaping was not associated with any reduction in perceived benefits or having any negative effects on perceived day-to-day functioning or ability to enjoy life, so there is no evidence of any negative effect of regular vaping post-quitting on well-being. Coupled with the positive associations, it suggests that there are more likely to be net benefits on well-being of continuing to vape after smoking cessation. That all said, the effect sizes are modest and the significance levels marginal, so replication is needed before any stronger conclusions can be made. We thus conservatively conclude that there are no differential experienced short- or medium-term adverse effects of regular vaping on mental well-being over complete cessation of nicotine use post-quitting, and weak evidence of small positive effects on coping. We draw no conclusions about any causal relationship for reported changes.

Finally, the findings of widespread residual worry about future health are a cause of some concern. Levels of worry seemed to be most common in 40- to 54-year-old smokers, particularly in relation to those aged 55 and older. The 40s and early 50s is the period when the risk of major health harms is increasing rapidly, but before the peak in premature mortality, so it might represent some concern about recovery from unobserved effects, which may be less likely for those older and for whom more signs of adverse effects may have already been present. It would be interesting to conduct qualitative research on possible differential experiences post-quitting by age with a focus on understanding whether perceived harm already caused (albeit minor) might influence perceptions after quitting. Our theorizing is that continued worry may act as a risk factor for relapse, as the reassurance of the benefit of quitting is at least partly reduced. There is thus a need to test whether worry is an independent predictor of relapse.

It is notable that around a quarter of the ex-smokers reported at least one adverse effect on their well-being a year or more after quitting. This indicates a high level of persistence in the face of some costs. The group who suffer adverse effects may require different forms of assistance than those who do not. Work is required to help find ways to minimize or reverse these effects. That the more common negative effects were on the emotional coping measures and were less common among “daily vaping quitters” suggests that vaping may be a means to reduce these negative effects for some at least. This is an area that deserves more focused research to see if vaping is indeed protective, and if so, whether its protective effects can be increased.

This study has a number of limitations that restrict what we can reasonably conclude about the impact of the duration of time quit smoking on post-quitting experiences. First, being cross-sectional, it is not designed to show causal relationships. Measures like time quit cannot be manipulated experimentally, and the trends we found are suggestive of adaptive processes reducing negatives and marginally increasing positives of experiences over time, which is consistent with what is known about adaptation more generally. This also applies to the vaping findings, as noted above. Related to this, our findings are independent of the respondents’ imputed causality for any effects they have observed, as we asked only about change, so do not know if the differences we found, especially for vaping, are imputed as due to vaping or not. Second, at least part of the observed effect of time quit is likely due to differential attrition. Ex-smokers who have negative experiences are more likely to relapse [10,11]. Longitudinal studies are needed to see what changes occur within individuals. If poor initial outcomes persist, there are different implications from if they are likely to reduce with time. Third, any findings regarding vaping versus not vaping should be interpreted with caution, since those who self-select to vape are different from those who do not, and we did not ask the vapers in this study whether they believe the experienced effects would have been greater or smaller if they had not continued to vape. There is evidence that those who take up vaping may be overall more dependent on nicotine [24], which might suggest that the apparent small benefits of vaping after quitting might have been partly masked here, as those more dependent were less likely to report benefits, but as our attempts to control for this made little difference, we think this unlikely to be a major issue. Future studies, including randomized trials, could help to assess how persistent nicotine vaping impacts both the positive and negative experiences associated with cessation of smoking.

## 5. Conclusions

In conclusion, most ex-smokers reported at least one area of improvement in their well-being, and perceived benefits of smoking cessation were higher with longer duration of time quit smoking, especially for those quit for more than a few months. There is little evidence that regular vaping post-quitting reduces any of these desirable experiences, and weak evidence that regular vaping after-quitting smoking is associated with im-proved emotion coping. Quitting smoking is a positive experience for most in terms of aspects of well-being, but some experience no benefits and those experiencing negative changes may need additional supports to overcome these problems.

## Figures and Tables

**Table 1:** Sample characteristics, by duration of time quit.

	Overall (*n* = 1379)	0–3 Months % (*n* = 213) #	4 Months–1 Year % (*n* = 312)	1–2 Years % (*n* = 322)	2–5 Years % (*n* = 532)	Significance
**Total**	1379	15.6	22.5	22.3	39.5	
**Country**						
Canada	450	15.1	24.2	24.7	36.0	χ^2^(9) = 10.7
US	371	18.1	18.3	21.6	42.1	*p* = 0.29
England	359	13.9	25.6	23.4	37.1	
AU	199	14.1	21.6	23.6	40.7	
**Vaping status**						
Current daily	374	10.7	21.9	22.5	44.9	χ^2^(9) = 20.8
Current weekly	53	18.9	35.9	18.9	26.4	*p* = 0.014
Past regular	321	16.2	21.5	26.2	36.1	
Never regular	631	17.6	22.5	22.8	37.1	
**NRT status**						
Daily user	57	40.4	15.8	19.3	24.6	χ^2^(3) = 28.4
Other	1319	14.4	22.9	23.5	39.2	*p* < 0.001
**Sex**						
Male	621	14.3	23.8	22.2	39.6	χ^2^(3) = 2.6
Female	758	16.4	21.6	24.3	37.3	*p* = 0.46
**Age**						
18–24	148	25.0	33.4	35.1	6.1	χ^2^(9) = 85.0
25–39	295	14.9	23.1	24.8	37.3	*p* = 0.001
40–54	362	12.7	24.9	20.7	41.7	
55+	574	15.0	18.1	21.3	45.6	
**Education**						
Low	338	14.5	17.5	25.2	42.9	χ^2^(6) = 11.1
Moderate	631	16.8	23.1	22.0	38.0	*p* = 0.085
High	410	14.2	26.1	23.9	35.9	
**Income**						
Low	369	17.3	21.7	26.0	35.0	χ^2^(9) = 10.1
Moderate	402	13.9	25.1	23.4	37.6	*p* = 0.34
High	530	14.5	21.5	22.5	41.5	
Not reported	78	20.5	21.8	16.7	41.0	

# Row percentages; in some analyses the numbers were less than the total due to missing cases.

**Table 2:** Correlations among measures of perceived effects of quitting.

	Stress Coping	Negative Affect Coping	Enjoyment of Life	Daily Functioning
**Stress coping**				
Negative affect coping *	**0.69**			
	1379			
	*p* < 0.001			
**Enjoyment of life**	**0.36**	**−0.00**		
	1379	1379		
	*p* < 0.001	*p* < 0.001		
**Daily functioning**	**0.42**	**0.43**	**0.47**	
	844	844	844	
	*p* = 0.006	*p* = 0.973	*p* = 0.001	
**Persisting worry**	**0.05**	**−0.00**	**0.09**	**0.12**
	1377	1377	1377	844
	*p* = 0.006	*p* = 0.973	*p* = 0.001	*p* < 0.001

* The values are Pearson r correlations, observations and significance; and this applies to other measures.

**Table 3:** Perceived effects of quitting, by duration of time quit and vaping status.

			Duration of Time Quit				Vaping Daily	
	Overall (*n* = 1379) *n*	Overall, %	0–3 Months, %	4 Months–1 Year, %	1–2 Years, %	2–5 Years, %	Vaping Quitters, %	Other Quitters, %
**Stress coping**			χ^2^(6) = 19.2, *p* = 0.004				χ^2^(2) = 25.3, *p* < 0.001	
Improved	333	24.1	20.2	22.8	28.6	23.9	22.5	25.2
Same	800	58.0	53.1	59.3	54.7	61.3	67.4	54.5
Worsened	246	17.8	26.8	18.0	16.8	14.9	10.2	20.7
**Negative affect coping**			χ^2^(6) = 20.4, *p* = 0.004				χ^2^(2) = 22.8, *p* < 0.001	
Improved	329	23.9	16.9	21.5	29.8	24.4	21.9	24.6
Same	825	59.8	59.6	61.2	55.9	61.5	68.7	56.5
Worsened	225	16.3	23.5	17.3	14.3	14.1	9.4	18.9
**Enjoyment of life**			χ^2^(6) = 28.5, *p* < 0.001				χ^2^(2) = 2.5, *p* = 0.33	
Improved	661	47.9	40.9	45.5	48.1	52.1	50.5	47.0
Same	647	46.9	47.4	51	46.9	44.4	45.5	47.5
Worsened	71	5.2	11.7	3.5	5.0	3.6	4.0	5.6
**Daily functioning**			χ^2^(4) = 11.9, *p* = 0.026				χ^2^(2) = 3.9, *p* = 0.14	
Improved	475	56.3	47.4	58.6	59.9	NA	55.6	56.5
Same	313	37.1	42.7	35.9	34.5	NA	40.5	35.6
Worsened	56	6.6	9.9	5.5	5.6	NA	3.9	7.5
**Persisting worry**			χ^2^(9) = 12.1, *p* = 0.207				χ^2^(3) = 7.6, *p* = 0.05	
Not at all worried	215	15.6	11.7	18.0	18.3	14.2	14.2	16.2
A little worried	619	45.0	44.1	43.6	40.7	48.7	46.3	44.5
Moderately worried	345	25.1	26.7	25.0	24.8	23.1	28.6	23.7
Very worried	198	14.4	17.4	13.5	16.2	14.4	11.0	15.7

In some analyses, the numbers were less than the total due to missing cases. NA: not applicable.

**Table 4:** Associations between perceived effects of quitting smoking and length of time quit and other variables—ordered logistic regression results.

Factor	Stress Coping (1. Worsened; 2. Same; 3. Improved) Adjusted OR (95% CI) #	Negative Affect Coping (1. Worsened; 2. Same; 3. Improved) Adjusted OR (95% CI) #	Enjoyment of Life (1. Worsened; 2. Same; 3. Improved) Adjusted OR (95% CI) #	Daily Functioning (1. Worsened; 2. Same; 3. Improved) Adjusted OR (95% CI) #	Persisting Worry (1. Not at All; 2. A Little; 3. Moderate; 4. very Worried) Adjusted OR (95% CI) #
**Length of time quit**					
0–3 months	Ref	Ref	Ref	Ref	Ref
4 months–1 year	1.18 (0.82–1.70)	1.11 (0.77–1.61)	1.27 (0.88–1.83)	1.29 (0.91–1.83)	0.82 (0.58–1.17)
1–2 years	1.42 (0.98–2.04)	1.53 (1.06–2.22) *	1.37 (0.94–1.99)	1.48 (1.04–2.10)*	0.92 (0.65–1.31)
2–5 years	1.26 (0.89–1.79)	1.29 (0.90–1.84)	1.48 (1.04–2.12)*	NA	0.89 (0.63–1.24)
**Vaping status**					
Daily (Vaping quitters)	1.30 (1.01–1.66) *	1.28 (0.99–1.65)	1.10 (0.86–1.42)	0.97 (0.71–1.33)	0.98 (0.77–1.25)
Other quitters	Ref	Ref	Ref	Ref	Ref
**Country**					
Canada	Ref	Ref	Ref	Ref	Ref
US	0.87 (0.66–1.16)	0.92 (0.60–1.23)	0.71 (0.43–0.79) *	0.82 (0.57–1.16)	0.72 (0.54–0.95) *
England	0.66 (0.49–0.89) *	0.67 (0.49–0.90) **	0.58 (0.53–0.95) ***	0.52 (0.37–0.74) ***	0.76 (0.57–1.01)
Australia	0.70 (0.49–1.01)	0.68 (0.47–0.97) *	0.68 (0.48–0.98) *	0.85 (0.56–1.30)	0.61 (0.44–0.87) **
**Sex**					
Male	Ref	Ref	Ref	Ref	Ref
Female	0.72 (0.58–0.90) **	0.73 (0.59–0.92) **	1.00 (0.80–1.24)	1.24 (0.96–1.62)	1.52 (1.22–1.88) ***
**Age**					
18–24	1.17 (0.76–1.79)	1.16 (0.76–1.79)	0.99 (0.65–1.52)	1.27 (0.81–1.97)	1.03 (0.68–1.56)
25–39	1.09 (0.80–1.49)	1.08 (0.79–1.47)	0.96 (0.71–1.31)	1.32 (0.91–1.92)	1.27 (0.95–1.71)
40–54	1.21 (0.92–1.58)	1.19 (0.90–1.56)	1.43 (1.08–1.89) *	1.49 (1.05–2.10) *	1.43 (1.10–1.87) **
55+	Ref	Ref	Ref	Ref	Ref
**Cigarettes per day at peak in the past**					
1–9	Ref	Ref	Ref	Ref	Ref
10–19	0.65 (0.48–0.88) **	0.61 (0.45–0.83) **	1.04 (0.77–1.41)	0.96 (0.69–1.34)	1.15 (0.86–1.54)
20+	0.69 (0.50–0.95) **	0.67 (0.49–0.92) *	1.18 (0.86–1.62)	1.09 (0.75–1.57)	1.29 (0.95–1.74)
**Perceived past health effects of smoking**					
Not at all	Ref	Ref	Ref	Ref	Ref
Just a little	1.55 (1.08–2.23)	1.28 (0.89–1.84)	1.95 (1.36–2.79) ***	1.29 (084–1.97)	4.76 (3.27–6.92) ***
A fair amount	2.12 (1.44–3.13) **	1.70 (1.15–2.51) **	2.62 (1.78–3.87) ***	2.46 (1.54–3.91)***	18.93 (12.51–28.65) ***
A great deal	2.58 (1.63–4.10) ***	1.49 (0.93–2.38)	2.11 (1.32–3.37) **	2.46 (1.41–4.31) **	84.89 (51.03–141.24) ***
Don’t know	1.07 (0.71–1.63)	0.91 (0.60–1.38)	1.10 (0.72–1.67)	0.80 (0.49–1.33)	4.43 (2.89–7.68)***
**Strength of urges to smoke**					
Not felt	Ref	Ref	Ref	Ref	Ref
Slightly	0.51 (0.38–0.68) ***	0.55 (0.41–0.74) ***	0.69 (0.52–0.93) *	0.71 (0.52–0.98) *	1.13 (0.86–1.49)
Substantially	0.47 (0.30–0.72) ***	0.35 (0.22–0.53) ***	0.77 (0.50–1.18)	0.42 (0.27–0.68) **	1.41 (0.93–2.12)
**NRT use**					
Daily	0.98 (0.55–1.72)	0.78 (0.44–1.36)	0.83 (0.47–1.45)	1.00 (0.52–1.91)	1.21 (0.70–2.09)
All others	Ref	Ref	Ref	Ref	Ref

# The full models contain all the variables listed in the table, plus education and income. Because education and income were largely unrelated to capacity to enjoy life and other perceived effects of quitting, their adjusted odds ratios (ORs) and confidence intervals (CIs) are not reported in the table. * Significant at *p* < 0.05, ** at *p* < 0.01, *** *p* < 0.001. Ref = reference value. For “country” we treated Canada as the reference because it is the most extreme country, making interpretation of results easiest. “Daily vaping quitters” were less likely to report that their ability to cope with stress worsened (10.2%) compared to “Other quitters” (20.7%; χ^2^ = 25.3, *p* < 0.001). A similar difference was found for coping with negative feelings (9.4% vs. 18.9%, respectively; χ^2^ = 22.8, *p* < 0.001). There were no significant differences by vaping status for changes in capacity to enjoy life (*p* = 0.325) or day-to-day functioning (*p* = 0.141), but the non-significant trend favored “Daily vaping quitters” over “Other quitters” for day-to-day functioning (see Table 3). Vaping status was also unrelated to persisting worry.

## Data Availability

The data are jointly owned by a third party in each country that collaborates with the International Tobacco Control Policy Evaluation (ITC) Project. Data from the ITC Project are available to approved researchers 2 years after the date of issuance of cleaned data sets by the ITC Data Management Centre. Researchers interested in using ITC data are required to apply for approval by submitting an International Tobacco Control Data Repository (ITCDR) request application and subsequently to sign an ITCDR Data Usage Agreement. The criteria for data usage approval and the contents of the Data Usage Agreement are described online (http://www.itcproject.org, accessed on 20 July 2022). The authors of this paper obtained the data following this procedure. This is to confirm that others would be able to access these data in the same manner as the authors. The authors did not have any special access privileges that others would not have.

## License Statement:

I, the submitting author, have the right to grant and does grant on behalf of all authors of the work an exclusive license and/or a nonexclusive license for contributions from authors.
